# The Early Aftermath of the Türkiye Earthquake: A Plastic Surgeon's View

**DOI:** 10.1055/a-2077-1924

**Published:** 2023-05-29

**Authors:** Bulent Sacak

**Affiliations:** 1 Department of Plastic Reconstructive and Aesthetic Surgery, Marmara University School of Medicine, İstanbul, Türkiye; 2 Secretary General, Turkish Society of Plastic Reconstructive and Aesthetic Surgery, İstanbul, Türkiye

**Figure:**
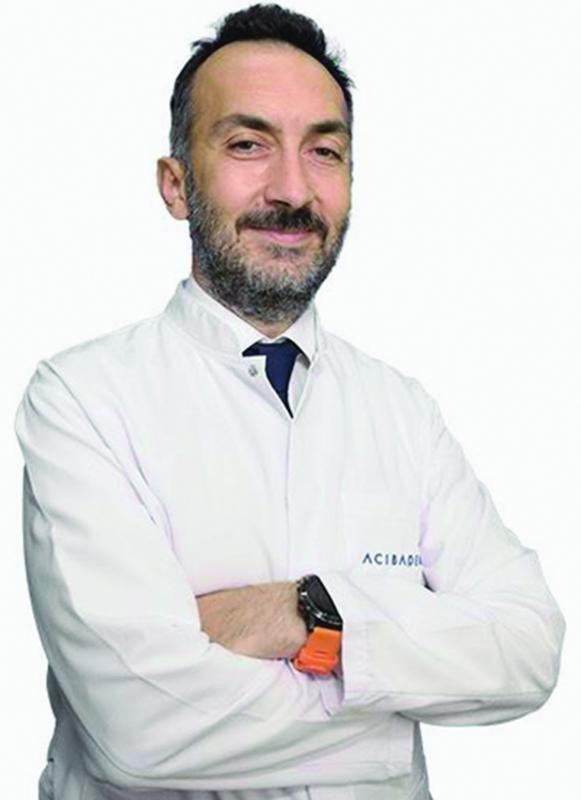
Bulent Sacak, MD

An ordinary patient visits in the morning in our department. The next patient has a crush injury and is requiring reconstructive surgery on her lower extremity. We listen to how the patient's injury occurred. She is one of the many victims of the earthquake, and she was rescued after spending 30 hours under the rubble. She lost her family, home, and all her possessions. She is the only member of her family who was in the room at the time of the earthquake to be pulled alive from the rubble. She has waited to be rescued side by side with her relatives who already lost their lives. It is not difficult to understand that the clear agony in her eyes is not caused by her wounds, yet it is impossible not to show pity and a strong compassion. She is one of the thousands of patients recovered from the rubble and hospitalized almost in every hospital in Türkiye. I try to hide my almost tearful eyes from the patient and my colleagues, but I am sure I am not the only one.

Approximately a half month before the day these lines were written, on the early morning of February 6, a 7.8 magnitude earthquake struck Southeast Türkiye and northern Syria. In addition to many aftershocks on the same day, the region was completely destroyed by a 7.7 magnitude earthquake a few hours later, on a second fault line in the same region. The republic, celebrating its centennial in 2023, faced the greatest destruction and disaster it has ever seen.

For today, according to official records, the number of people who lost their lives in Türkiye alone has exceeded 40,000 and the injured are over 100,000. Considering that the impact area of the earthquake extends for approximately 330 km and the population in this region is over 10 millions, it is not easy to estimate not only the number of people who have passed away or have been injured, but also the extent of suffering and agony due to the disaster.

It would be impossible, as a human being or as a plastic surgeon, to remain indifferent to such a destruction. Turkish Society of Plastic, Reconstructive and Aesthetic Surgery immediately started to assign voluntary members to the field in an organized manner in the first hours after the earthquake. During the first 4 days, teams of four or five plastic surgeons were voluntarily assigned to the region to work in field hospitals in harmony with the ministry of health and disaster coordination units. The first teams arriving to the region provided emergency medicine services such as patient triage, resuscitation, and first care rather than plastic surgery procedures.

In the second stage we are in, the patients in the field hospitals have been transferred to the hospitals mostly in the immediate proximity of the region, resulting in large concentrations in these hospitals. For these patients, the need for plastic surgery for operations such as wound debridement, fasciotomy, and limb-sparing interventions have reached a very high level as expected. The society is closely monitoring the supplies and manpower in these hospitals in simultaneous communication with 26 hospitals in 12 provinces and making an effort to provide voluntary plastic surgeons and missing materials to the institutions in a coordinated manner. The number of voluntary members of our society working in the region is close to 50 surgeons so far. Our efforts are just a drop in the ocean. Almost everyone, every institution from home and abroad are trying to do their best to help to the region and to send humanitarian and financial aid to the region. We are witnessing a solidarity and cooperation in such a scale that we have never seen before. I hereby would sincerely like to thank all our friends, colleagues, and sister societies, who sent messages from abroad and offered their support and assistance. Once again, solidarity has been proven as the key to getting through such a period.

We have lost too many people. In one way, it strongly feels like the meaning of fundamental human emotions such as pain and grief has changed forever. But on the other hand, we who are left behind have to find ways to stand up and move on. Our patient is on her bed, desperately waiting for her needs for medical attention to be met. We have been trained to overcome the everyday emotions we face. We have been trained to close the most impossible wounds. We are proud to be plastic and reconstructive surgeons, being able to directly touch and make a difference in the lives of patients even in these times. We have an indefinite time ahead of us to recover somehow completely. If recovery is possible, it will only be possible because of the merits we have earned through our training and our exceptional solidarity.

